# Involvement of the vagus nerve in the anorectic effect of monoacylglycerol acyltransferase 2 inhibition in mice

**DOI:** 10.1002/osp4.693

**Published:** 2023-07-06

**Authors:** Kosuke Takemoto, Hideaki Kato, Kenichi Higashino

**Affiliations:** ^1^ Drug Discovery & Disease Research Laboratory Shionogi & Co., Ltd. Osaka Japan; ^2^ Laboratory of Veterinary Pathology Joint Faculty of Veterinary Medicine Yamaguchi University Yamaguchi Japan

**Keywords:** anorectic effect, MGAT2, oleoylethanolamide, vagus nerve

## Abstract

**Background:**

Many of the drugs used for obesity treatment have adverse effects on the central nervous system. Therefore, novel treatments, such as peripherally acting drugs, are needed. Monoacylglycerol acyltransferase 2 (MGAT2), highly expressed in the small intestine, catalyzes the first step of triacylglycerol re‐synthesis. MGAT2 inhibition suppresses food intake in high‐fat diet (HFD)‐fed mice, but the mechanisms remain unclear. Here, the involvement of the vagus nerve in MGAT2 inhibition‐induced feeding suppression was investigated.

**Methods:**

Fasted mice were administered an MGAT2 inhibitor. Food intake was measured after HFD re‐feeding, and the effect of capsaicin pretreatment on changes in food intake was evaluated. The number of c‐fos‐positive cells in the nucleus tractus solitarius and levels of appetite regulators were determined after HFD re‐feeding or lipid gavage.

**Results:**

The anorectic effect of the MGAT2 inhibitor was abolished when vagus nerve function was interrupted by capsaicin. MGAT2 inhibition increased the number of c‐fos‐positive cells in the nucleus tractus solitarius and elevated intestinal oleoylethanolamide, plasma peptide tyrosine–tyrosine and plasma glucagon‐like peptide‐1 levels.

**Conclusion:**

MGAT2 inhibition suppresses feeding behavior via peripheral vagus nerve signaling and may serve as a novel anti‐obesity strategy with a low risk of unexpected central nervous system‐related adverse effects.

## Graphical abstract

MGAT2 inhibitor–induced feeding suppression was counteracted by systemic capsaicin pretreatment. C‐fos expression in the nucleus tractus solitarius and jejunal oleoylethanolamide (OEA) content were increased in MGAT2 inhibitor–treated mice under high‐fat diet feeding conditions. These findings indicate that MGAT2 inhibition may suppress feeding behavior via vagus nerve signaling and that an increased level of OEA is associated with loss of appetite.

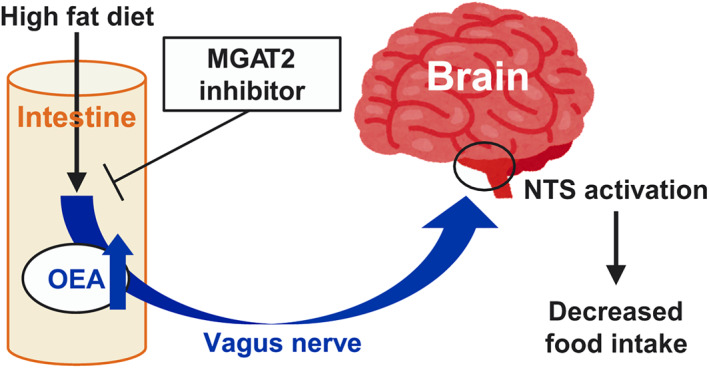

## 1 INTRODUCTION

Many drugs developed for the treatment of obesity regulate appetite by directly stimulating the central nervous system because excessive food intake leads to obesity.^1^ However, several centrally acting anti‐obesity drugs have been withdrawn from the market owing to unacceptable adverse effects such as increased risk of cardiovascular events and suicidal ideation.^2^, ^3^ Some other centrally acting drugs also have adverse effects such as increased risk of elevated blood pressure or nausea/vomiting.^4^, ^5^, ^6^ Therefore, peripherally acting drugs, which are assumed to have a low risk of central nervous system‐related adverse effects, may be attractive treatment agents.

Monoacylglycerol acyltransferase 2 (MGAT2) is highly expressed in the small intestine and involved in the absorption of dietary triacylglycerol (TAG) in the small intestine.^7^, ^8^, ^9^ TAG is hydrolyzed by pancreatic TAG lipase to unesterified fatty acid (FA) and 2‐monoacylglycerol (2‐MAG) in the lumen of the intestine.^9^, ^10^ Digested lipids are taken up by the proximal small intestine and re‐synthesized to TAG.^9^, ^10^ The re‐synthesized TAG then enters the circulation in the form of chylomicrons.^9^, ^10^ MGAT, the key enzyme mediating intestinal TAG re‐synthesis, catalyzes the diacylglycerol synthesis reaction using FA‐coenzyme A and 2‐MAG, and MGAT2 plays a dominant role in intestinal MGAT activity in mice and humans.^9^, ^11^, ^12^

Mice deficient in MGAT2 have the same phenotype as wild‐type mice under standard chow diet feeding conditions but are resistant to diet‐induced obesity under high‐fat diet (HFD) feeding conditions.^8^ The anti‐obesity effects of MGAT2 inhibition are at least partially attributable to feeding suppression because MGAT2 deficiency decreases HFD intake in mice.^13^ In MGAT2‐deficient mice, the plasma level of anorectic glucagon‐like peptide‐1 (GLP‐1) is elevated.^8^, ^13^ In addition, administration of 2‐oleoyl glycerol (2‐OG), an MGAT2 substrate, increases the plasma GLP‐1 concentration.^14^ These findings suggest that increased plasma GLP‐1 level is associated with the anorectic effect of MGAT2 inhibition. However, the mechanisms underlying the changes in feeding behavior remain largely unclear.

By transmitting nutrient‐derived signals, the peripheral vagus nerve plays a crucial role in regulating food intake.^15^ Dietary TAG is digested in the small intestine, and the digested lipids induce the production of appetite regulators such as oleoylethanolamide (OEA), peptide tyrosine–tyrosine (PYY), and GLP‐1.^16^, ^17^, ^18^ These appetite regulators activate the nucleus tractus solitarius (NTS), which is connected to the intestine mainly by the vagus nerve.^19^, ^20^, ^21^ The anorectic effects of the appetite regulators are ablated by truncal vagotomy or capsaicin pretreatment, which blocks the function of afferent vagal fibers.^19^, ^22^ As MGAT2 participates in the intestinal absorption of dietary TAG, a vagally mediated signal may contribute to reduced food intake following MGAT2 inhibition in mice.

The aim of this study was to investigate the involvement of the vagus nerve pathway in the anorectic effect of pharmacological MGAT2 inhibition in mice.

## 2 MATERIALS AND METHODS

### 2.1 Animals and drugs

Six‐week‐old male *C57BL/6* mice were obtained from CLEA Japan (Tokyo, Japan) and housed individually before experimental use. All mouse studies were approved by the Institutional Animal Care and Use Committee of Shionogi & Co., Ltd., and all experiments were performed at facilities accredited by the Association for Assessment and Accreditation of Laboratory Animal Care International.

The selective MGAT2 inhibitor used in this study was synthesized as previously reported,^23^ and the structure was confirmed using ^1^H nuclear magnetic resonance and liquid chromatography–mass spectrometry (Figure S1). The MGAT2 inhibitor was suspended in aqueous 0.5% w/v hydroxypropylmethylcellulose (Shin‐Etsu Chemical, Tokyo, Japan). The mice were orally administered the suspended MGAT2 inhibitor at a dose of 10 mg/kg body weight. The MGAT2 inhibitor dose that induced maximal inhibition of TAG absorption for at least 17 h was selected for experimental use (Figure S2). Experimental details on the analysis of TAG absorption can be found in the Supplemental Information S1.

### 2.2 Capsaicin treatment

To impair the function of the afferent vagus nerve, capsaicin was administered as previously described with a slight modification.^24^ The mice were fed a 60% HFD (58Y1; TestDiet, St. Louis, MO, USA) for more than 4 weeks and subcutaneously administered 20 mg/kg capsaicin (Sigma‐Aldrich, St. Louis, MO, USA) under anesthesia induced with 30 mg/kg pentobarbital. Two days after the first administration, the mice were administered 40 mg/kg capsaicin under the same conditions. Four days after the second administration, the mice were interperitoneally injected with 5 mg/kg capsaicin while conscious. Capsaicin was dissolved in 10% Tween 80 and 10% ethanol in saline. Control mice were subjected to the same experimental procedure described above, but without capsaicin administration. Before feeding studies, the mice were allowed to recover for more than 4 days after the last capsaicin injection. There was no difference in food intake between capsaicin‐treated and control mice. To confirm that the capsaicin treatment successfully blocked afferent vagal function, the anorectic effect of cholecystokinin‐8 (Peptide Institute Inc., Osaka, Japan) in capsaicin‐treated mice was analyzed. It was found that the vagally mediated satiety signal^25^, ^26^ was blunted (data not shown).

### 2.3 Measurement of food intake

Mice were fed the 60% HFD for more than 4 weeks, fasted overnight, administered the vehicle or MGAT2 inhibitor, and refed. Food intake was measured for 2 and 4 h after the start of re‐feeding. Experimental details under standard chow diet feeding conditions can be found in the Supplemental Information S1.

### 2.4 Measurement of c‐fos immunoreactivity

Mice were deeply anesthetized 2 h after the start of HFD re‐feeding and perfused transcardially with 4% paraformaldehyde in phosphate buffered saline (PBS). The brain tissue was sampled from the mice, post‐fixed in 4% paraformaldehyde solution, transferred to 30% sucrose in H_2_O, and embedded in OCT compound. Serial 17‐μm‐thick coronal sections were cut and heated for 15 min in the target retrieval solution (Agilent Technologies, Santa Clara, CA, USA) under microwave irradiation at 90°C for antigen retrieval. The sections were preincubated for 30 min in 0.3% hydrogen peroxide in methanol, blocked for 60 min in PBS containing 3% bovine serum albumin, and incubated overnight with anti‐c‐fos mouse monoclonal antibodies (sc‐271243; Santa Cruz Biotechnology, Dallas, TX, USA) diluted 1:600 in PBS containing 1% bovine serum albumin at 4°C. The sections then reacted for 30 min with Histofine Simple Stain MAX‐PO (Nichirei Biosciences, Tokyo, Japan) and visualized using a Histofine DAB substrate kit (Nichirei Biosciences). The number of c‐fos‐positive cells in the NTS and area postrema (bregma −7.32 to −7.76 mm) and in the arcuate nucleus and paraventricular nucleus (bregma −1.22 to −1.58 mm) was determined automatically using a BZ‐X700 instrument (Keyence, Osaka, Japan).

### 2.5 Analysis of appetite regulators and intestinal lipids

Intestinal tissues and blood were collected from mice under anesthesia induced with isoflurane 2 h after the start of re‐feeding. To determine OEA and 2‐OG levels, the intestinal tissues were homogenized in approximately 10 volumes of methanol containing 10 mM dibutyl‐hydroxytoluene and centrifuged at 20,000 × *g* for 5 min. The levels of OEA and 2‐OG in the supernatant extracted from the intestinal tissues were determined using liquid chromatography–mass spectrometry analysis with Nexera MP and LCMS‐8060 instruments (Shimadzu, Kyoto, Japan). To determine TAG and FA levels, the intestinal tissues were homogenized in 1 mL of H_2_O, mixed with 5 mL of chloroform/methanol (3:2, v/v) and 1 mL of 1 M NaCl solution, and centrifuged at 840 × *g* for 20 min to obtain two phases. The lower phase containing lipids was evaporated under a nitrogen stream, and the dried residue was dissolved in 1 mL of isopropanol. The levels of TAG and FA in the dissolved solution were determined using enzymatic methods with commercial kits (Sekisui Medical, Tokyo, Japan) with Hitachi 7170 autoanalyzer (Hitachi, Tokyo, Japan). The blood was centrifuged at 5000 × *g* for 10 min at 4°C to obtain plasma. The levels of PYY and total GLP‐1 in the plasma were determined using the mouse/rat PYY ELISA Kit (Wako Pure Chemical Industries, Osaka, Japan) and GLP‐1 ELISA Kit Wako High Sensitive (Wako Pure Chemical Industries), respectively. For lipid‐loading tests, the mice were fasted overnight, administered the vehicle or MGAT2 inhibitor, and administered 20% lipids (Intralipos Injection 20%; Otsuka Pharmaceutical Factory, Tokushima, Japan) at a dose of 10 mL/kg by oral gavage. One hour after the oral gavage, the intestinal tissues and blood were collected from the mice under anesthesia induced with isoflurane.

### 2.6 Statistical analysis

Data are presented as mean ± standard error of the mean (SEM). The results were analyzed using a two‐tailed Welch's *t*‐test or Tukey's multiple comparison test with SAS Version 9.4 for Windows (SAS Institute). Results with *p* < 0.05 were considered statistically significant.

## 3 RESULTS

### 3.1 Capsaicin pretreatment abrogated MGAT2 inhibition‐induced feeding suppression

**FIGURE 1:**
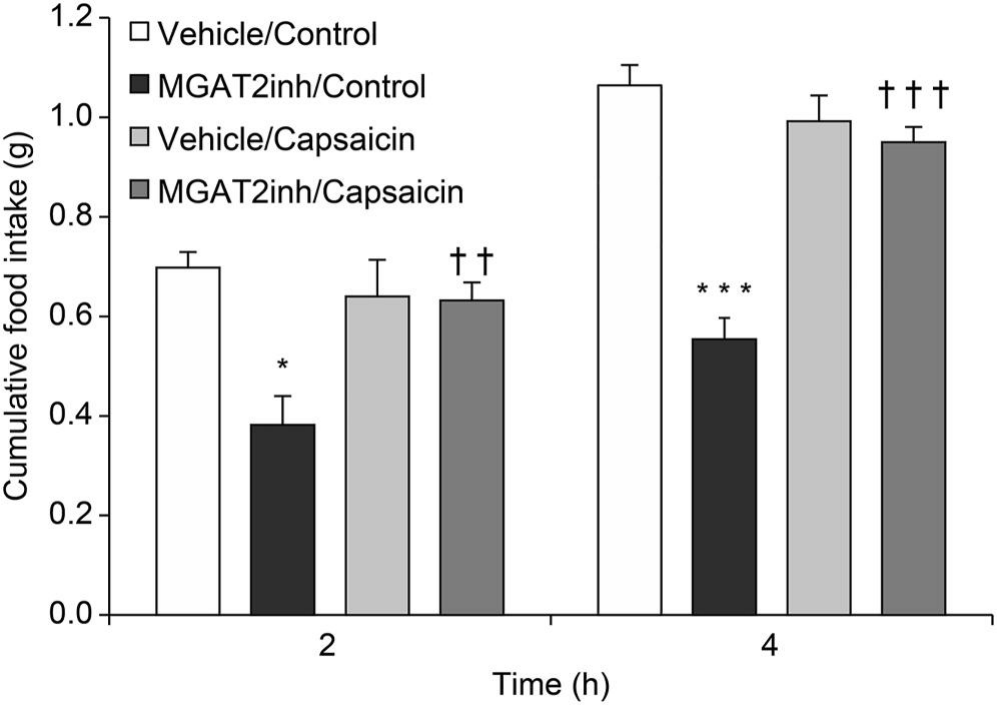
The effect of the MGAT2 inhibitor on feeding behavior in capsaicin‐treated mice after HFD re‐feeding. The MGAT2 inhibitor (MGAT2inh; 10 mg/kg) was orally administered to fasted mice. Food intake was measured for 2 and 4 h after the start of re‐feeding. The results are expressed as mean ± SEM (*n* = 5). *p < 0.05, ***p < 0.001 versus vehicle group, ^††^ *p* < 0.01, ^†††^ *p* < 0.001 versus the control group (Tukey's multiple comparison test). HFD, high‐fat diet; MGAT2, monoacylglycerol acyltransferase 2; SEM, standard error of the mean.

To confirm the involvement of the vagus nerve in MGAT2 inhibition‐induced feeding suppression, the effect of MGAT2 inhibition on feeding behavior was examined in capsaicin‐treated mice under HFD feeding conditions. With vehicle administration, the food intake did not differ between the capsaicin‐treated and control mice (Vehicle/Control vs. Vehicle/Capsaicin [2h], *p* > 0.99; Vehicle/Control vs. Vehicle/Capsaicin [4h], *p* = 0.89) (Figure 1). In comparison to the vehicle administration, the administration of a single dose of the MGAT2 inhibitor significantly decreased the food intake for 2 and 4 h after the start of HFD re‐feeding (Vehicle/Control vs. MGAT2inh/Control [2h], *p* < 0.05; Vehicle/Control vs. MGAT2inh/Control [4h], *p* < 0.001). These anorectic effects were abrogated in capsaicin‐treated mice (MGAT2inh/Control vs. MGAT2inh/Capsaicin [2h], *p* < 0.01; MGAT2inh/Control vs. MGAT2inh/Capsaicin [4h], *p* < 0.001) (Figure 1). Under standard chow diet feeding conditions, there was no decrease in food intake for 2 h after the start of re‐feeding in MGAT2 inhibitor‐treated mice (*p* = 0.24) (Figure S3A).

### 3.2 Activation of NTS neurons by MGAT2 inhibition

**FIGURE 2:**
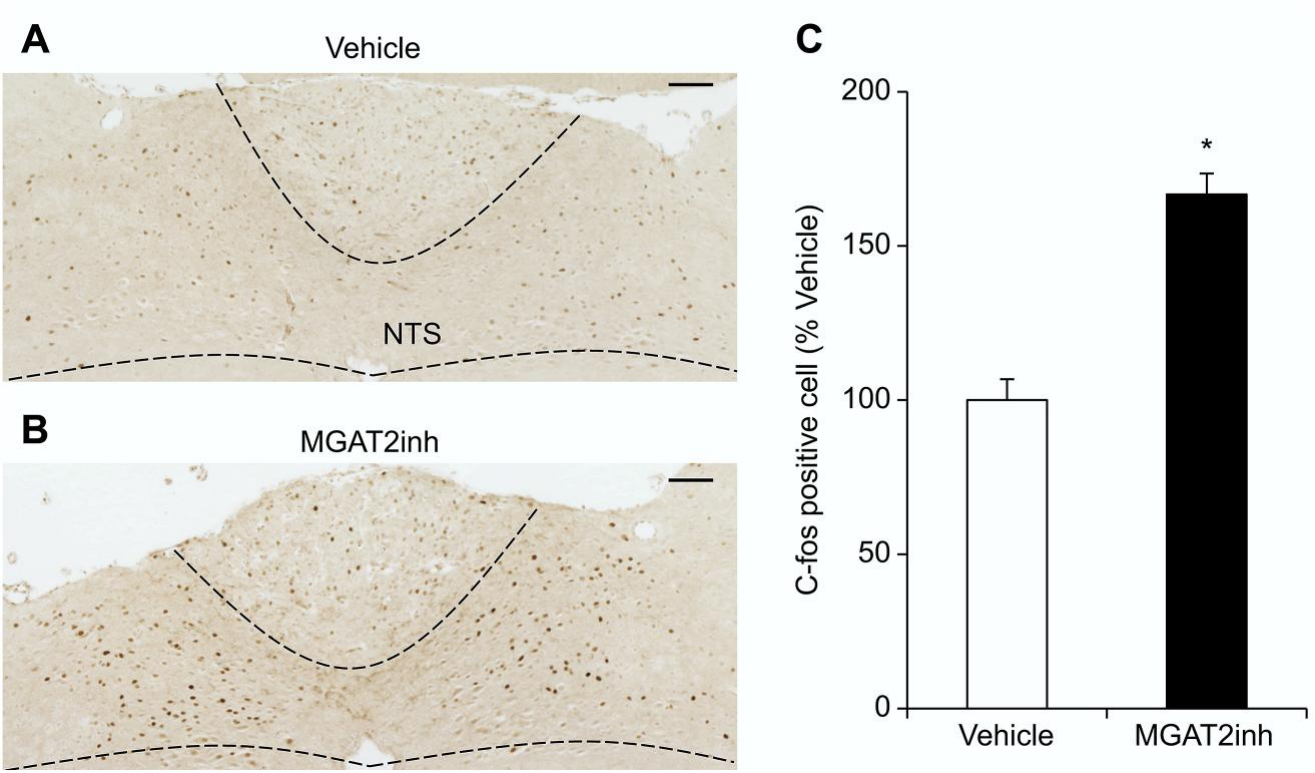
The effect of the MGAT2 inhibitor on c‐fos immunoreactivity in the NTS of mice after HFD re‐feeding ((A)–(C)). The MGAT2 inhibitor (10 mg/kg) was orally administered to fasted mice. The mice were euthanized 2 h after re‐feeding, and brain samples were collected. The number of c‐fos‐positive cells was counted automatically, and the results are presented as a percentage of that in the vehicle‐treated group and as mean ± SEM (*n* = 8). Scale bars represent 100 μm. *p < 0.05 versus vehicle group (Welch's *t*‐test). HFD, high‐fat diet; MGAT2, monoacylglycerol acyltransferase 2; NTS, nucleus tractus solitarius; SEM, standard error of the mean.

To investigate whether MGAT2 inhibition activates the afferent vagal pathway, the effect of MGAT2 inhibition on c‐fosTimmunoreactivity, as a marker for neuronal activation, was examined in the NTS of mice. The MGAT2 inhibitor‐treated mice exhibited a significantly increased number of c‐fos‐positive cells in the NTS 2 h after the start of HFD re‐feeding, compared with that in the vehicle‐treated mice (*p* < 0.05) (Figure 2A–C). The MGAT2 inhibitor treatment also increased the number of c‐fos‐positive cells in the area postrema, which has dense reciprocal connections with the NTS and influences appetite, compared with that in the vehicle‐treated mice (*p* < 0.01) (Figure S4A). C‐fos expression in hypothalamic appetite‐related regions, such as the arcuate nucleus and paraventricular nucleus, did not differ between the vehicle‐treated and MGAT2 inhibitor‐treated mice (arcuate nucleus, *p* = 0.27; paraventricular nucleus, *p* = 0.74) (Figure S4B, S4C).

### 3.3 MGAT2 inhibition‐induced changes in appetite regulator and intestinal lipid levels

**FIGURE 3:**
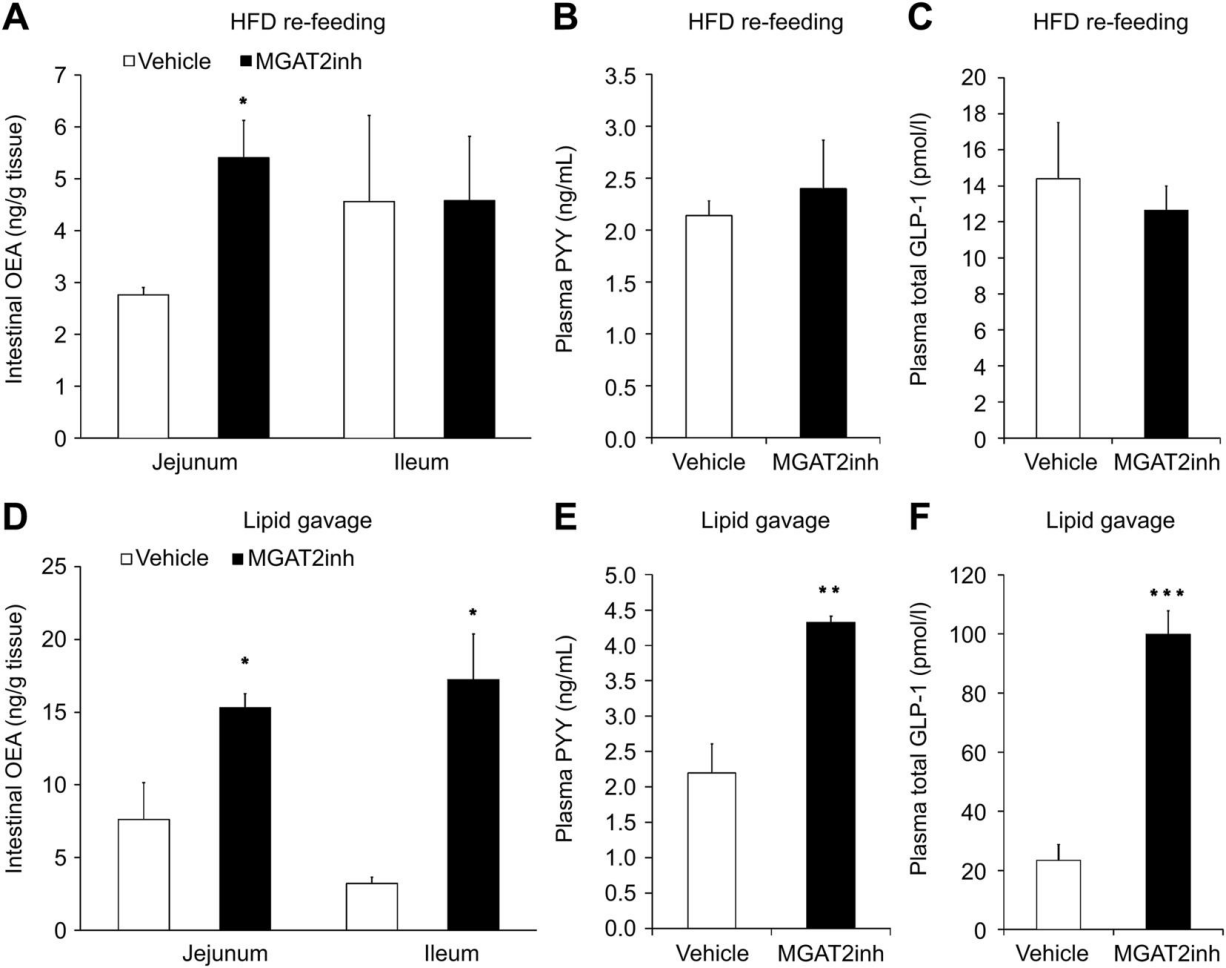
The effect of the MGAT2 inhibitor on the levels of intestinal OEA ((A) and (D)), plasma PYY ((B) and (E)), and plasma total GLP‐1 ((C) and (F)) in mice after HFD re‐feeding or lipid gavage. The MGAT2 inhibitor (10 mg/kg) was orally administered to fasted mice. The mice were euthanized 2 h after re‐feeding or 1 h after lipid gavage, and samples of intestinal tissue and plasma were collected. Intestinal levels of OEA were analyzed using liquid chromatography–mass spectrometry, and plasma levels of PYY and total GLP‐1 were analyzed using ELISA kits. The results are expressed as mean ± SEM ((A) *n* = 4; ((B)–(F)) *n* = 5). *p < 0.05, **p < 0.01, ***p < 0.001 versus vehicle group (Welch's *t*‐test). GLP‐1, glucagon‐like peptide‐1; HFD, high‐fat diet; MGAT2, monoacylglycerol acyltransferase 2; OEA, oleoylethanolamide; PYY, peptide tyrosine–tyrosine; SEM, standard error of the mean.

To explore the mechanisms by which MGAT2 inhibition results in feeding suppression, the effect of MGAT2 inhibition on the levels of appetite regulators that activate the afferent vagal pathway was examined in mice. The MGAT2 inhibitor significantly elevated the level of OEA in the jejunum but not in the ileum 2 h after the start of HFD re‐feeding compared with the vehicle (jejunal OEA, *p* < 0.05; ileal OEA, *p* = 0.86) (Figure 3A). The plasma PYY and total GLP‐1 levels after re‐feeding were not affected by MGAT2 inhibition (PYY, *p* = 0.64; total GLP‐1, *p* = 0.63) (Figure 3B,C). The effect of MGAT2 inhibition on the levels of the appetite regulators was also examined in mice administered a large amount of bolus lipid via oral gavage. The MGAT2 inhibitor significantly increased the jejunal and ileal levels of OEA and plasma levels of PYY and total GLP‐1 at 1 h post lipid gavage compared with the vehicle (jejunal OEA, *p* < 0.05; ileal OEA, *p* < 0.05; PYY, *p* < 0.01; total GLP‐1, *p* < 0.001) (Figure 3D–F).

**FIGURE 4:**
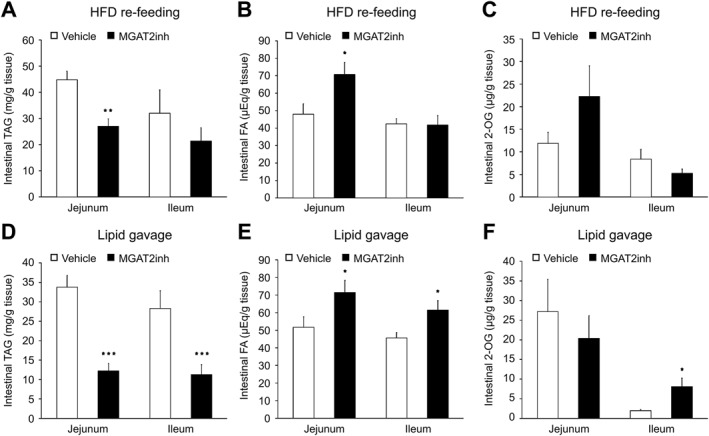
The effect of the MGAT2 inhibitor on the levels of intestinal TAG ((A) and (D)), FA ((B) and (E)), and 2‐OG ((C) and (F)) in mice after HFD re‐feeding or lipid gavage. The MGAT2 inhibitor (10 mg/kg) was orally administered to fasted mice. The mice were euthanized 2 h after re‐feeding or 1 h after lipid gavage, and intestinal tissue samples were collected. The levels of intestinal TAG and FA were determined using enzymatic methods, and the level of intestinal 2‐OG was determined using liquid chromatography–mass spectrometry. The results are expressed as mean ± SEM. ((A) and (B)) *n* = 8 ((C) and (F)) *n* = 5 ((D) and (E) *n* = 13). *p < 0.05, **p < 0.01***p < 0.001 versus vehicle group (Welch's *t*‐test). 2‐OG, 2‐oleoyl glycerol; FA, fatty acid; HFD, high‐fat diet; MGAT2, monoacylglycerol acyltransferase 2; SEM, standard error of the mean; TAG, triacylglycerol.

To further understand the relationship between MGAT2 inhibition and changes in appetite regulator levels, the effect of MGAT2 inhibition on intestinal lipid levels was examined in mice under the same conditions in which appetite regulators were measured. Two hours after the start of HFD re‐feeding, the MGAT2 inhibitor significantly decreased jejunal TAG level and increased jejunal FA level compared with the vehicle, whereas MGAT2 inhibition did not affect the jejunal level of 2‐OG and ileal levels of TAG, FA, and 2‐OG (jejunal TAG, *p* < 0.01; ileal TAG, *p* = 0.32; jejunal FA, *p* < 0.05; ileal FA, *p* = 0.89; jejunal 2‐OG, *p* = 0.21; ileal 2‐OG, *p* = 0.26) (Figures 4A–C). Conversely, the MGAT2 inhibitor significantly decreased the jejunal and ileal levels of TAG and increased the jejunal level of FA, but not that of 2‐OG, and ileal levels of FA and 2‐OG at 1 h post lipid gavage compared with the vehicle (jejunal TAG, *p* < 0.001; ileal TAG, *p* < 0.001; jejunal FA, *p* < 0.05; ileal FA, *p* < 0.05; jejunal 2‐OG, *p* = 0.52; ileal 2‐OG, *p* < 0.05) (Figures 4D–F). Two hours after the start of standard chow diet re‐feeding, the jejunal and ileal levels of TAG and FA were not affected by the MGAT2 inhibitor (jejunal TAG, *p* = 0.18; ileal TAG, *p* = 0.42; jejunal FA, *p* = 0.05; ileal FA, *p* = 0.56) (Figure S3B, S3C).

## 4 DISCUSSION

The aim of the present study was to clarify whether the anorectic effect of MGAT2 inhibition in mice was mediated by the vagus nerve. The selective pharmacological MGAT2 inhibition suppressed short‐term feeding behavior after HFD re‐feeding, and the anorectic effect of MGAT2 inhibition was abrogated by blocking the afferent vagus nerve via capsaicin pretreatment. Moreover, MGAT2 inhibition upregulated the expression of c‐fos in the NTS, which receives afferent input from the vagus nerve, and increased the jejunal OEA level after HFD re‐feeding. Following lipid gavage, MGAT2 inhibition elevated the plasma levels of PYY and total GLP‐1, which is in agreement with previous study findings,^8^, ^13^, ^27^, ^28^ and increased the jejunal and ileal levels of OEA. These appetite regulators inhibit feeding behavior through the afferent vagus nerve.^18^, ^22^ Based on these findings, MGAT2 inhibition is highly likely to suppress HFD intake in mice via the afferent vagus nerve and NTS signaling in response to increased levels of intestinal OEA, plasma PYY, and plasma GLP‐1.

The jejunal level of OEA, but not plasma levels of PYY and GLP‐1, was elevated by MGAT2 inhibition after HFD re‐feeding, and the elevation was accompanied by a decrease in the jejunal TAG level and an increase in the jejunal FA level. OEA is generated from oleic acid, a major FA, predominantly in the jejunum.^18^ As it limits the incorporation of oleic acid into TAG in enterocytes,^12^ MGAT2 inhibition may elevate the intestinal OEA level through an increase in the oleic acid level. Although other unknown appetite regulators may contribute to the anorectic effect of MGAT2 inhibition, these findings indicate that an increase in the level of OEA, rather than the levels of PYY and GLP‐1, is associated with decreased food intake in mice under HFD feeding conditions.

Unlike that after HFD re‐feeding, MGAT2 inhibition after oral administration of a large amount of bolus lipid elevated the levels of all three appetite regulators accompanied by a decrease in the jejunal and ileal TAG levels and an increase in the jejunal FA level and ileal FA and 2‐OG levels. Previous studies have shown that MGAT2 inhibition affects the spatial distribution of intestinal TAG and increases the levels of digested lipids, such as FA and 2‐MAG, in the distal small intestine of mice after bolus lipid administration.^8^, ^27^ PYY and GLP‐1 are secreted by L‐cells distributed in the distal small intestinal mucosa in response to FA and 2‐OG.^23^, ^29^, ^30^ After bolus lipid administration, excessive lipid‐induced lipid influx into the distal small intestine may trigger an increase in the ileal FA and 2‐OG levels and the subsequent enhancement of plasma PYY and GLP‐1 secretion in MGAT2 inhibitor‐treated mice.

The results suggest that MGAT2 is a potential target for regulating appetite via peripheral nerve stimulation. Several centrally acting anti‐obesity drugs have unacceptable central nervous system‐related adverse effects, and, in contrast to pharmacological therapy, Roux‐en‐Y gastric bypass decreases calorie intake and has strong anti‐obesity effects without neurologic and psychiatric adverse events.^1^, ^2^, ^3^, ^31^, ^32^, ^33^, ^34^, ^35^ Roux‐en‐Y gastric bypass involves rerouting of the lipid absorption pathway to the distal small intestine, resulting in increased OEA, GLP‐1, and PYY levels and decreased fat intake in rodents.^36^, ^37^ These phenotypes are strikingly similar to those induced by pharmacological MGAT2 inhibition. The Roux‐en‐Y gastric bypass and MGAT2 inhibition‐induced reduction in food intake is transient and abolished after long‐term HFD feeding.^23^, ^38^ Although the detailed effects of MGAT2 inhibition on the central nervous system and on long‐term weight loss through transient food intake reduction remain unclear, MGAT2 inhibition may be an attractive, non‐invasive therapeutic strategy for regulating appetite, with a low risk of unexpected central nervous system‐related adverse effects.

In summary, this study revealed that MGAT2 inhibition decreases food intake in mice via the vagus nerve. The findings suggest that the increase in the level of OEA following MGAT2 inhibition is a key mechanism underlying vagal activation and the subsequent feeding suppression. Although further studies are required to clarify the detailed mechanisms of chronic pharmacological MGAT2 inhibition, our findings may inform the development of MGAT2‐targeting therapeutic drugs for obesity and obesity‐related diseases.

## CONFLICT OF INTEREST STATEMENT

All authors are employees of Shionogi & Co., Ltd.

## Supporting information

Supporting Information S1
