# Supplementary material for: Involvement of the vagus nerve in the anorectic effect of monoacylglycerol acyltransferase 2 inhibition in mice
Source: Obes Sci Pract. 2023 Jul 6;9(6):601–8. doi: 10.1002/osp4.693 (PMC10712405; doi:10.1002/osp4.693)
Supplement: Supplementary file 1 — Supporting Information S1 [file OSP4-9-601-s001.docx]

**Involvement of the vagus nerve in the anorectic effect of monoacylglycerol acyltransferase 2 inhibition in mice**

Kosuke Takemoto^1,2^, Hideaki Kato^1^, Kenichi Higashino^1^

^1^Drug Discovery & Disease Research Laboratory, Shionogi & Co., Ltd., Osaka, Japan

^2^Laboratory of Veterinary Pathology, Joint Faculty of Veterinary Medicine, Yamaguchi University, Yamaguchi, Japan

**CORRESPONDENCE:** Kosuke Takemoto

Drug Discovery & Disease Research Laboratory, Shionogi & Co., Ltd., Shionogi Pharmaceutical Research Center

3-1-1, Futaba-cho, Toyonaka, Osaka 561-0825, Japan

E-mail: [kosuke.takemoto@shionogi.co.jp](mailto:kosuke.takemoto@shionogi.co.jp)

**SUPPLEMENTARY EXPERIMENTAL PROCEDURES**

**Analysis of the inhibitory effect on TAG absorption**

Mice were administered the vehicle or MGAT2 inhibitor and fasted overnight. At 13 h after the administration, the mice were intraperitoneally injected with 500 mg/kg Pluronic F-127 and then administered 10 mL/kg of 50 µCi/kg [carboxyl-^14^C] triolein in corn oil via oral gavage. The mice were anesthetized with isoflurane, and blood was collected approximately 17 h after the administration of the MGAT2 inhibitor. Plasma radioactivity was quantified by measuring ionizing radiation using the MicroBeta TRILUX counter (Perkin Elmer, Waltham, MA, USA).

**Analysis under standard chow diet feeding conditions**

Six-week-old male *C57BL/6* mice were maintained on standard chow diet (CE-2, 12% kcal fat) and fasted overnight, administered the vehicle or MGAT2 inhibitor, and re-fed. Food intake was measured for 2 h after the start of re-feeding. The intestinal tissues were collected from mice under anesthesia induced with isoflurane 2 h after the start of re-feeding. The intestinal levels of TAG and FA were determined in the same manner as described in the main text.

**SUPPLEMENTARY FIGURES**

**

**

**Figure S1.** Chemical structure of the selective MGAT2 inhibitor used in this study.

**

**

**Figure S2.** Plasma radioactivity after oral gavage of [^14^C] TAG in the MGAT2 inhibitor-treated groups. The MGAT2 inhibitor was orally administered to mice prior to oral gavage of corn oil containing 50 µCi/kg [^14^C] TAG. Blood was collected approximately 17 h after the administration of the MGAT2 inhibitor, and plasma radioactivity was quantified by measuring ionizing radiation. The results are presented as a percentage of plasma radioactivity in the vehicle-treated group and as mean ± SEM (n = 5). ^***^*P* < 0.001 vs. vehicle group (Dunnett's multiple comparison test).





**Figure S3.** Effect of the MGAT2 inhibitor on feeding behavior (A) and the levels of intestinal TAG (B) and FA (C) in mice after standard chow diet re-feeding. The MGAT2 inhibitor (10 mg/kg) was orally administered to fasted mice. Food intake was measured for 2 h after the start of re-feeding. The mice were euthanized 2 h after re-feeding, and the intestinal tissues were collected. The results are expressed as mean ± SEM (n = 5).

**

**

**Figure S4.** Effect of the MGAT2 inhibitor on c-fos immunoreactivity in the area postrema (A), arcuate nucleus (B), and paraventricular nucleus (C) of mice. The MGAT2 inhibitor (10 mg/kg) was orally administered to fasted mice. The mice were euthanized 2 h after re-feeding, and brain samples were dissected. The number of c-fos-positive cells was counted automatically, and the results are presented as a percentage of that in the vehicle-treated group and as mean ± SEM (n = 8). ***P* < 0.01 vs. Vehicle group (Welch’s *t*-test).
